# Rare genetic variation in fibronectin 1 (*FN1*) protects against *APOEε4* in Alzheimer’s disease

**DOI:** 10.1007/s00401-024-02721-1

**Published:** 2024-04-10

**Authors:** Prabesh Bhattarai, Tamil Iniyan Gunasekaran, Michael E. Belloy, Dolly Reyes-Dumeyer, Dörthe Jülich, Hüseyin Tayran, Elanur Yilmaz, Delaney Flaherty, Bengisu Turgutalp, Gauthaman Sukumar, Camille Alba, Elisa Martinez McGrath, Daniel N. Hupalo, Dagmar Bacikova, Yann Le Guen, Rafael Lantigua, Martin Medrano, Diones Rivera, Patricia Recio, Tal Nuriel, Nilüfer Ertekin-Taner, Andrew F. Teich, Dennis W. Dickson, Scott Holley, Michael Greicius, Clifton L. Dalgard, Michael Zody, Richard Mayeux, Caghan Kizil, Badri N. Vardarajan

**Affiliations:** 1 Department of Neurology, Columbia University Irving Medical Center, Columbia University New York, New York, NY USA; 2 Taub Institute for Research on Alzheimer’s Disease and the Aging Brain, Columbia University Irving Medical Center, Columbia University, New York, NY USA; 3 Gertrude H. Sergievsky Center, College of Physicians and Surgeons, Columbia University, New York, NY USA; 4 Department of Neurology and Neurological Sciences, Stanford University, Stanford, CA USA; 5 NeuroGenomics and Informatics Center, Washington University School of Medicine, St. Louis, MO USA; 6 Department of Neurology, Washington University School of Medicine, St. Louis, MO USA; 7 Department of Molecular, Cellular, and Developmental Biology, Yale University, New Haven, CT 06520 USA; 8 Department of Pathology and Cell Biology, Columbia University Irving Medical Center, New York, NY 10032 USA; 9 Henry M. Jackson Foundation for the Advancement of Military Medicine, Bethesda, MD 20817 USA; 10 Quantitative Sciences Unit, Department of Medicine, Stanford University, Stanford, CA USA; 11 Department of Medicine, College of Physicians and Surgeons, Columbia University New York, New York, USA; 12 School of Medicine, Pontificia Universidad Catolica Madre y Maestra, Santiago, Dominican Republic; 13 Department of Neurology, CEDIMAT, Plaza de la Salud, Santo Domingo, Dominican Republic; 14 School of Medicine, Universidad Pedro Henriquez Urena (UNPHU), Santo Domingo, Dominican Republic; 15 Department of Neuroscience, Mayo Clinic Florida, Jacksonville, FL 32224 USA; 16 Department of Neurology, Mayo Clinic Florida, Jacksonville, FL 32224 USA; 17 Department of Anatomy, Physiology and Genetics, Uniformed Services University of the Health Sciences, Bethesda, MD 20814 USA; 18 The American Genome Center, Center for Military Precision Health, Uniformed Services University of the Health Sciences, Bethesda, MD USA; 19 New York Genome Center, New York, NY 10013 USA; 20 Department of Psychiatry, College of Physicians and Surgeons, Columbia University, 1051 Riverside Drive, New York, NY 10032 USA; 21 Department of Epidemiology, Mailman School of Public Health, Columbia University, 722 W 168th St., New York, NY 10032 USA

## Abstract

The risk of developing Alzheimer’s disease (AD) significantly increases in individuals carrying the *APOEε4* allele. Elderly cognitively healthy individuals with *APOEε4* also exist, suggesting the presence of cellular mechanisms that counteract the pathological effects of *APOEε4*; however, these mechanisms are unknown. We hypothesized that *APOEε4* carriers without dementia might carry genetic variations that could protect them from developing *APOEε4-*mediated AD pathology. To test this, we leveraged whole-genome sequencing (WGS) data in the National Institute on Aging Alzheimer's Disease Family Based Study (NIA-AD FBS), Washington Heights/Inwood Columbia Aging Project (WHICAP), and Estudio Familiar de Influencia Genetica en Alzheimer (EFIGA) cohorts and identified potentially protective variants segregating exclusively among unaffected *APOEε4* carriers. In homozygous unaffected carriers above 70 years old, we identified 510 rare coding variants. Pathway analysis of the genes harboring these variants showed significant enrichment in extracellular matrix (ECM)-related processes, suggesting protective effects of functional modifications in ECM proteins. We prioritized two genes that were highly represented in the ECM-related gene ontology terms, *(FN1)* and collagen type VI alpha 2 chain (*COL6A2*) and are known to be expressed at the blood–brain barrier (BBB), for postmortem validation and in vivo functional studies. An independent analysis in a large cohort of 7185 *APOEε4* homozygous carriers found that rs140926439 variant in *FN1* was protective of AD (OR = 0.29; 95% CI [0.11, 0.78], *P* = 0.014) and delayed age at onset of disease by 3.37 years (95% CI [0.42, 6.32], *P* = 0.025). The FN1 and COL6A2 protein levels were increased at the BBB in *APOEε4* carriers with AD. Brain expression of cognitively unaffected homozygous *APOEε4* carriers had significantly lower FN1 deposition and less reactive gliosis compared to homozygous *APOEε4* carriers with AD, suggesting that FN1 might be a downstream driver of *APOEε4*-mediated AD-related pathology and cognitive decline. To validate our findings, we used zebrafish models with loss-of-function (LOF) mutations in *fn1b*—the ortholog for human *FN1*. We found that fibronectin LOF reduced gliosis, enhanced gliovascular remodeling, and potentiated the microglial response, suggesting that pathological accumulation of FN1 could impair toxic protein clearance, which is ameliorated with *FN1* LOF. Our study suggests that vascular deposition of FN1 is related to the pathogenicity of *APOEε4*, and LOF variants in FN1 may reduce *APOEε4*-related AD risk, providing novel clues to potential therapeutic interventions targeting the ECM to mitigate AD risk.

**Supplementary Information:**

The online version contains supplementary material available at 10.1007/s00401-024-02721-1.

## Introduction

Alzheimer’s disease (AD) is typically characterized clinically by progressive memory impairment and decline in other cognitive domains; however, there is a long pre-symptomatic period without clinical manifestations [74]. At death, pathological hallmarks in the brain include extracellular β-amyloid protein in diffuse and neuritic plaques and neurofibrillary tangles made of hyper-phosphorylated tau protein. AD, a progressive neurodegenerative disorder, is currently unpreventable, and, with available drugs only marginally affecting disease severity and progression, remains effectively untreatable. A critical barrier to lessening the impact of late-onset AD (LOAD) is the slow development of drugs that prevent or treat AD due, in part, to an incomplete characterization of the basic pathologic mechanisms. Determining which genes and gene networks contribute to AD could reveal the biological pathways for drug development and inform the development of genetic testing methods for identifying those at greatest risk for AD.

The presence of the *APOEε4* allele is among the most prominent genetic risk factors for AD in White, non-Hispanic populations [21], but the associated risks observed in African-Americans and Hispanics are somewhat lower [82]. Relative risk of AD associated with a single copy of *APOEε4* is 2.5- to 3.5-fold in Caucasians compared to 1.0–2.4 and 1–1.9 in African-Americans and Hispanics, respectively [9, 82]. However, in every population, homozygosity for the *APOEε4* allele is associated with increased risk and nearly complete penetrance [7, 64, 91]. *APOE*, a critical player in lipid metabolism and transport, has been extensively studied for its role in Alzheimer's disease (AD) and other neurodegenerative disorders [14, 55, 56]. The *APOEε4* allele is a well-established risk factor for late-onset AD, with carriers of this allele exhibiting an increased susceptibility to cognitive decline and dementia and earlier age at onset of clinical symptoms. However, within the population of *APOEε4* carriers, there is variability in age of onset and severity of AD symptoms. Some "resilient" or "cognitively normal, unaffected" individuals who carry the *ε4* allele do not develop AD or experience a delayed onset of symptoms. Several potential factors might contribute to the variability in AD risk and presentation among *APOEε4* carriers. Genetic modifier mutations outside of the *APOE* gene might interact with *APOEε4* to influence the risk of AD. *APOEε4* carriers might also be influenced by other risk factors for AD, such as vascular health, inflammation, and metabolic conditions. Interactions between *APOEε4* and these factors could modify the course of the disease. Certain rare protective variants in other genes could offset the risk posed by *APOEε4.*

Amid the well-documented association between *APOEε4* and AD risk, a growing body of evidence suggests intriguing nuances in the effects of this allele, particularly in certain subsets of individuals who defy the expected trajectory of cognitive decline and remain remarkably resilient to neurodegenerative diseases. Notably, heterozygosity of *APOEε4* has incomplete penetrance [31], and the polygenic risk of the rest of the genome could stratify *APOEε4* carriers into high- and low-risk strata. In this study, we aimed to identify putative protective mechanisms, influenced by genetic modifiers that might counteract the detrimental effects of the *APOEε4* allele. We sought to identify “protective” genetic factors that can modify or reduce the effect of *APOEε4* on AD risk and to identify new pathogenic mechanisms, proteins, and pathways that inform development of therapeutic targets and diagnostics.

## Results

### Whole-genome sequencing identifies putative protective variants in cognitively unaffected elderly *APOEε4* carriers

**Fig. 1:**
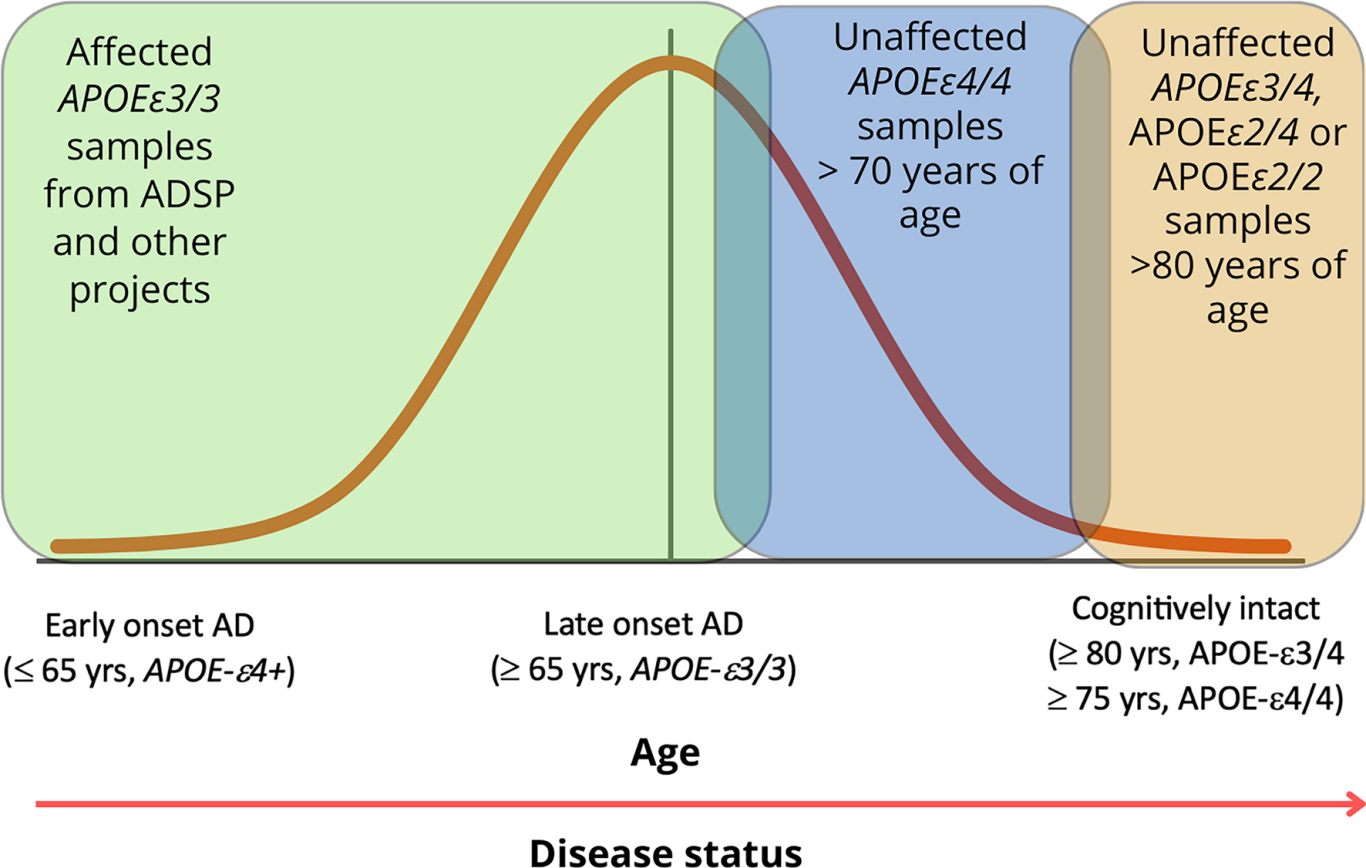
Study design. Comparison of the genomes of elderly *APOEε4* carriers with non-carriers

**Fig. 2:**
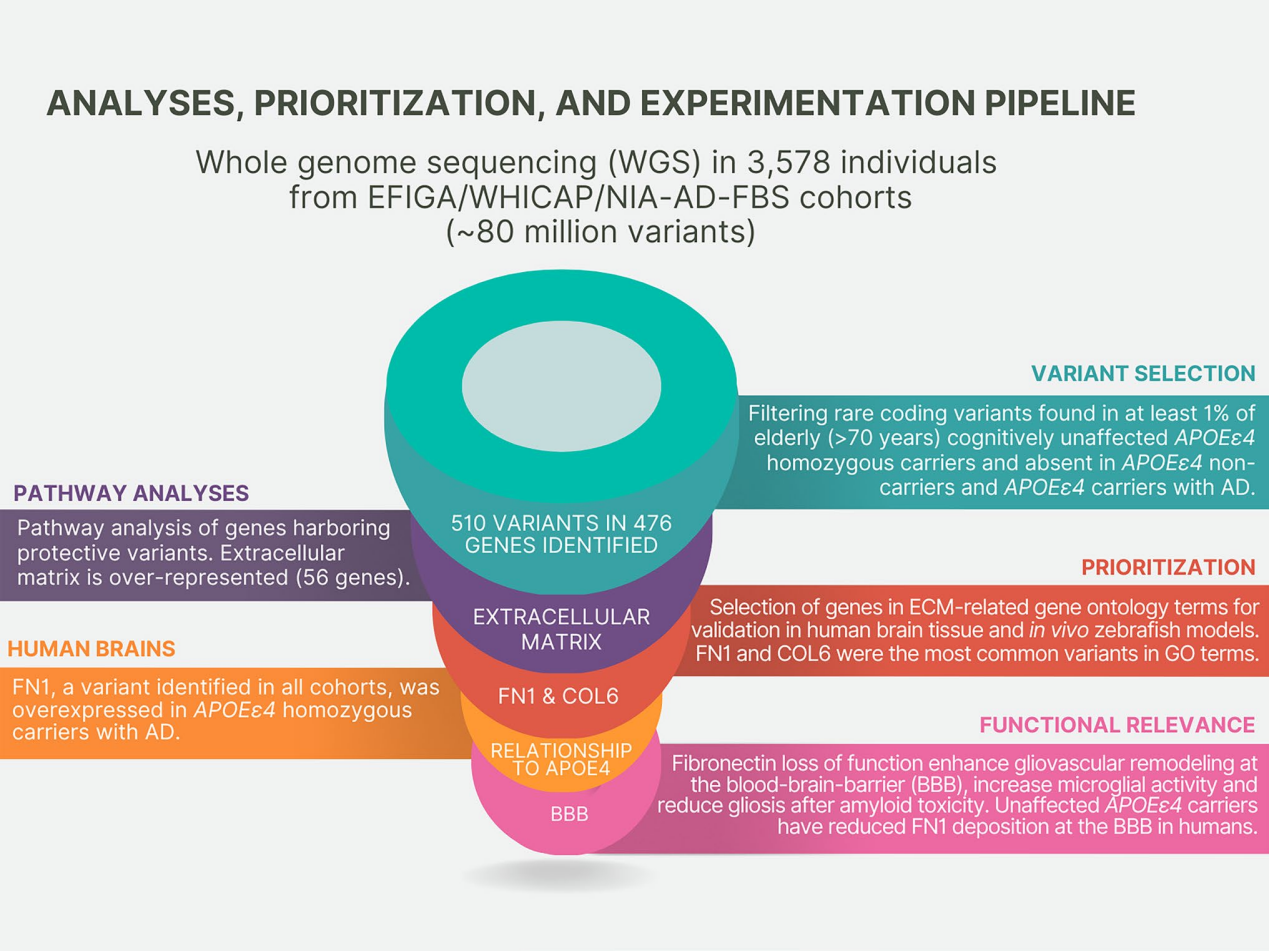
Schematic analytical pipeline for this study

**Fig. 3:**
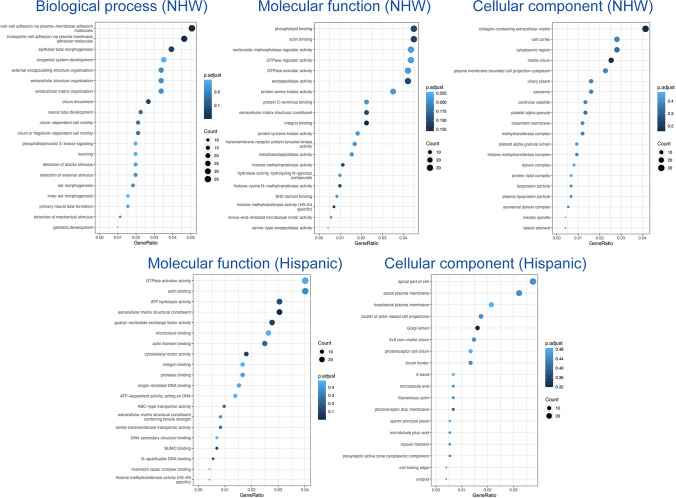
Pathway analysis of variants segregating in *APOEε4* carriers

We accessed whole-genome sequencing data in 3,578 individuals from over 700 non-Hispanic White and Caribbean Hispanic families multiply affected by AD (Table 1). After harmonization and QC of the WGS data, we identified rare (MAF < 1% in gnomAD) coding variants in the healthy elderly *APOEε4* homozygous (over the age of 70) and heterozygous (over the age of 80) carriers that were absent in non-carriers (Fig. 1). We further prioritized exon coding variants in healthy *APOEε4* carriers that bear the potential to be damaging to the resulting protein product. Supplementary Tables 1–3 provide the lists of candidate variants that were identified in cognitively unaffected elderly *APOEε4* carriers. Our strategy and analysis pipeline are summarized in Fig. 2. We found 510 variants in 476 genes that were present in at least 1% of *APOEε4* unaffected homozygous carriers (388 in EFIGA/WHICAP and 130 in NIA-AD FBS and 8 variants found in both datasets) (Supplementary Table 1 and 2). Two variants (rs116558455 and rs140926439) in the *FN1* gene (fibronectin-1) were found in healthy elderly *ε4* homozygous carriers in EFIGA/WHICAP and NIA-AD FBS cohorts with MAF = 1.85% and 3.33%, respectively (Table 2). In Hispanics, rs116558455 was absent in all *APOEε4* carriers with AD. In non-Hispanic Whites rs140926439 was absent in homozygous *APOEε4* AD patients, but found in 1% of heterozygous patients. Pathway analysis of the genes harboring variants segregating in *APOEε4* carriers identified several biological pathways and molecular functions such as “actin binding”, “microtubule binding”, and “extracellular matrix structural constituent” (Fig. 3). These results suggested a strong correlation with cellular morphologies and the architectural organization of those cells.

**Table 1:** Demographics of samples sequenced

	Hispanics	Non-Hispanic White
*N*	1840	590
AD cases	693	455
AD controls	1147	135
*Families with 2 or more individuals*		
# *APOEε4* heterozygotes	724	438
# *APOEε4* homozygotes	189	190
# *APOEε4* heterozygote AD cases	442	265
# *APOEε4* homozygote AD Cases	114	155
# *APOEε4* heterozygote healthy controls	282	161
# *APOEε4* homozygote healthy controls	75	30
# *APOEε4* homozygote healthy controls > = 70 years of age	27	15
# *APOEε4* heterozygote healthy controls > 80 years of age	75	45

**Table 2:** *FN1* minor allele frequencies

Cohorts	SNP	MAF in elderly* cognitively unaffected *APOEε4* homozygotes (%)	MAF in all cognitively unaffected *APOEε4* homozygotes (%)	MAF in *APOEε4* homozygous AD patients (%)	MAF in cognitively unaffected elderly* *APOEε4* heterozygotes (%)	MAF in all healthy *APOEε4* heterozygotes (%)	MAF in *APOEε4* heterozygous AD patients (%)
EFIGA and WHICAP	rs116558455	1.85	0.67	0.00	0.67	0.18	0.00
NIA-AD FBS	rs140926439	3.33	5.17	0.00	2.22	1.55	0.96

*Elderly *APOEε4* homozygous are over 70 years old and heterozygous are over 80 years old

### Potential protective alleles against *APOEε4* enrich extracellular matrix components

To determine the molecular mechanisms enriched in the protective alleles that we identified, gene ontology review was performed with term analyses for biological processes, cellular compartments, and molecular functions (Fig. 3). We found a strong enrichment for extracellular matrix (ECM)-related processes such as cell adhesion, ECM organization, integrin binding, and structural component of the ECM (Fig. 3). This suggested that functional alterations in the ECM composition could act as a protective mechanism in *APOEε4* carriers, both heterozygotes and homozygotes without dementia. We hypothesized that *APOEε4*-related increase in ECM components could be counteracted by loss-of-function (LOF) variants in those genes, leading to protection through rescue of pathological mechanisms that those ECM components partake.

To test our hypothesis, we selected two genes from the variant lists that were common in ECM-related gene ontology classes (Fig. 3), collagen type VI alpha 2 chain (*COL6A2*) and fibronectin 1 (*FN1*). These genes are well-known ECM components that harbor putatively protective variants in *APOEε4* cognitively unaffected carriers. Additionally, prioritized variants in FN1 were, respectively, present in both Hispanic and non-Hispanic White cohorts (Supplementary Table 1, Supplementary Table 2). *COL6A2* variation (rs777822883) generates a substitution of arginine at the 862nd residue to tryptophan, while *FN1* variation (rs140926439) converts the glycine at the 357th position to glutamic acid. Since both alterations result in change in charged residues (loss in COL6A2, gain in FN1), we hypothesized that these variations could have detrimental effect on the protein function, as charged interactions are essential for matric proteins and their stability [20, 61, 94]. Therefore, we analyzed the AlphaFold structures of these proteins in Ensembl (http://www.ensembl.org) and found that both variations are potentially detrimental according to SIFT, REVEL, and MetaL R predictions (Supplementary Fig. 1). Arginine in *COL6A2* at the 862nd position may coordinate with valine 859 and glutamic acid 858 in the alpha helix structure, while glycine at the 357th position in *FN1* may provide structural stability by coordinating with glutamic acid 358 and serine 355 (Supplementary Fig. 1). Therefore, we categorized these variants as likely loss-of-function alleles based on loss of electrostatic interactions.

### *FN1* variant is protective in an independent cohort of *APOEε4* carriers

We queried an independent collection of AD-related genetic cohorts from several sources: the Alzheimer’s Disease Genetic Consortium (ADGC), the Alzheimer’s Disease Sequencing Project (ADSP), and United Kingdom Biobank (UKB) resources, primarily consisting of non-Hispanic White individuals of European ancestry (EU) (Supplementary Table 4 and 5). A total of 465,669 NHW case–control individuals ages 60 and above were available after genetic and phenotypic quality control. Since rs116558455 is very rare in EU (gnomAD non-Finnish EU allele frequency = 0.016%), we focused on rs140926439 which is less rare in EU and thus provides sufficient allele counts to enable replication analyses (gnomAD non-Finnish EU allele frequency = 0.46%). We specifically focused analyses on *APOEε4*/4 carriers ages 60 and above (*N* = 7185), to interrogate whether there were any elevated frequencies for the rs140926439 minor allele (T) associated with reduced AD risk and delayed age at onset.

**Fig. 4:**
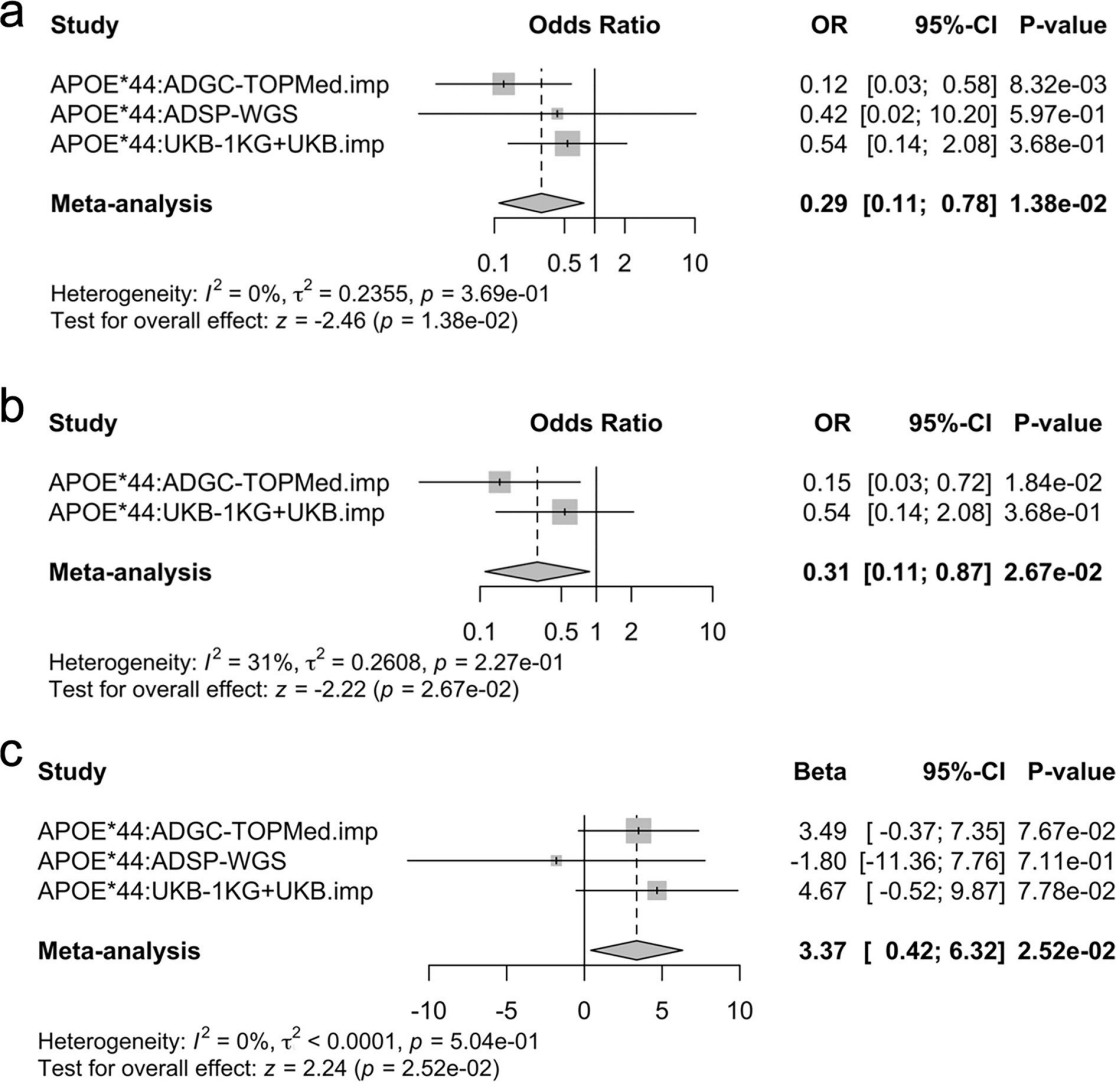
Replication analyses. **a** Forest plot showing the association of rs140926439 with Alzheimer’s disease risk in *APOEε4/4* carriers. Significance was considered at *P* < 0.05. Results across the datasets were combined using fixed-effects inverse-variance weighted meta-analysis. Cochran’s Q test indicated no significant heterogeneity. OR, odds ratio; CI, confidence interval. **b** Case–control regression sensitivity analyses for rs140926439 in *APOEε4/4* carriers. To ensure an independent replication of discovery findings, in ADGC, samples from NIA-AD FBS cohort were excluded and ADSP whole-genome sequencing data was fully excluded. Results across the datasets were combined using fixed-effects inverse-variance weighted meta-analysis. Cochran’s *Q* test indicated no significant heterogeneity. **c** Age at onset analyses for rs140926439 in *APOEε4/4* carriers. The large confidence intervals in ADSP whole-genome sequencing (WGS) individuals reflect that there were only two case carriers and one of those had an age at onset at 60 years (an outlier compared to other case carriers). Results across the datasets were combined using fixed-effects inverse-variance weighted meta-analysis. Cochran’s *Q* test indicated no significant heterogeneity

The variant rs140926439 was associated with strongly reduced risk of AD in *APOEε4/4* carriers (OR = 0.29; 95% CI [0.11, 0.78], *P* = 0.014; Table 3, Fig. 4a). Sensitivity analyses were conducted, which ensured that any overlapping samples with our discovery were excluded and corroborated primary findings (OR = 0.31; 95% CI [0.11, 0.87], *P* = 0.027; Supplementary Table 6, Fig. 4b). Secondary age at onset analyses further showed a significant protective effect in *APOEε4/4* carriers, delaying age at onset by 3.4 years for a single copy of the minor allele (beta = 3.37; 95% CI [0.42, 6.32], *P* = 0.025; Fig. 4c). These analyses represent the largest-to-date genetic association tests in a sample of *APOEε4/4* carriers at an age range relevant to AD.

**Table 3:** Replication of *FN1* variant in the ADSP and UKBB cohorts

Cohort	Total no.	Total, EAF (%)	CN, carrier no./total no. (%)	AD, carrier no./total no. (%)	CN–AD, EAF (%)	OR [95% CI]	*P*-value	CN, carrier age, mean (SD)	AD, carrier age, mean (SD)
ADGC	1510	0.43	4/129 (3.10%)	9/1381 (0.65%)	1.55–0.33%	0.12 [0.03, 0.58]	8.3E−03	78.8 (11.1)	72.9 (7.5)
ADSP	356	0.42	1/81 (1.23%)	2/275 (0.73%)	0.62–0.36%	0.42 [0.02, 10.2]	0.60	74.0 (–)	65.5 (7.8)
UKB	5319	0.54	54/4804 (1.12%)	3/515 (0.58%)	0.56–0.29%	0.54 [0.14, 2.08]	0.37	65.2 (4.4)	78.7 (2.9)
Combined	7185	0.51	59/5014 (1.18%)	14/2171 (0.64%)	0.59–0.32%	0.29 [0.11, 0.78]	0.014	66.3 (5.5)	73.1 (6.6)

### FN1 deposition correlates with APOEε4 dosage

**Fig. 5:**
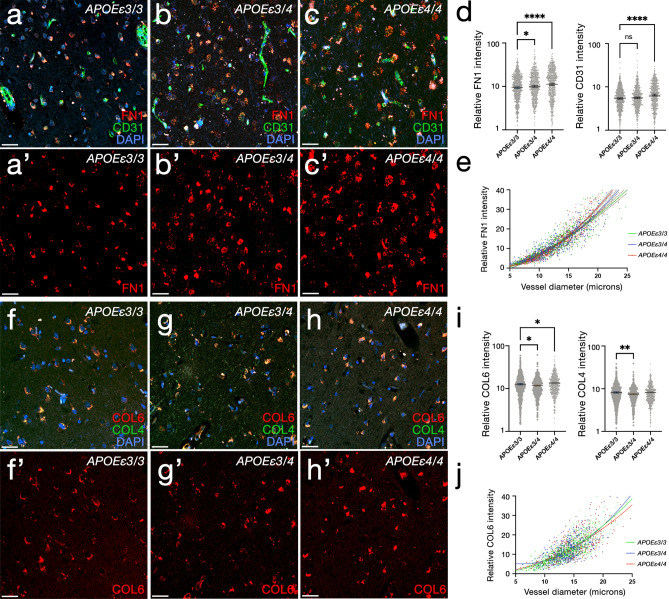
Changes in FN1 and COL6A2 according to *APOE* genotype. **a**–**c**′ Double IFS for CD31 (green) and FN1 (red) with DAPI nuclear counterstain in *APOEε3/3* (**a, a′**), *APOEε3/4* (**b, b′**) and *APOEε4/4* (**c, c′**). **d** FN1 and CD31 intensity comparisons in 2,044 blood vessels from 28 individuals. **e** Regression model for FN1 intensity with respect to blood vessel diameter in three *APOE* genotypes. **f–h′** Double IFS for COL4 (green) and COL6A2 (red) with DAPI nuclear counterstain in *APOEε3/3* (**f, f′**), *APOEε3/4* (**g, g′**) and *APOEε4/4* (**h, h′**). **i** COL4 and COL6A2 intensity comparisons in 1,816 blood vessels from 28 individuals. **j** Regression model for COL6A2 intensity with respect to blood vessel diameter in three *APOE* genotypes. Scale bars equal 100 μm

Based on our findings, we hypothesized that *APOEε4* dosage might correlate with deposition of COL6A2 and FN1, at the blood–brain barrier (BBB) basement membrane, one of their prominent expression locations, as FN1 is an important signaling molecule that interacts with specific integrins [69] expressed in various vascular niche cell types [52]. We immunostained and analyzed the brains of 27 individuals with known *APOE* genotypes (8 *APOEε4*/4 homozygous carriers with AD, 8 *APOEε3/4* heterozygote carriers with AD, and 11 *APOEε4* non-carriers (*APOEε3/3*) with AD (Supplementary Table 7) for FN1 and CD31 (endothelial cell marker), and COL6A2 and COL4 (a vascular basement membrane marker) (Fig. 5, Supplementary Dataset 1, Supplementary Dataset 2). We found that FN1 levels (Fig. 5a–c′) significantly increased with *APOEε4* dosage (Fig. 5d, Supplementary Fig. 2). Compared to *APOEε3/3* individuals, FN1 expression increased significantly in *APOEε3/4* (8.1%, *P* = 3.4e−02) and in *APOEε4 homozygous* individuals (26.6%, *P* = 3.1e−09). Least squares linear regression and non-linear fit comparison of FN1 intensities according to the diameter of the vessels showed that compared to *APOEε3/3*, FN1 expression is more prominent with increasing vessel size in *APOEε3/4* and *APOEε4/4* individuals (adjusted *R*^2^: *APOEε*3/3: 0.81, *APOEε3/4*: 0.86, *APOEε4/4*: 0.89; all *P* values are less than 1.0xE-15 for non-zero significance of the slopes) (Fig. 5e). Immunostainings for COL6 (Fig. 5f–h′) showed a non-linear relationship between *APOEε4* dosage and COL6 expression. *APOEε4* heterozygotes showed reduced (7.7%, *P* = 9.9e−03) homozygotes indicating increased levels of COL6 (6.7%, *P* = 3.4e−02) (Fig. 5i). COL4 expression is only reduced in *APOEε4* heterozygotes (8.6%, *P* = 1.4e−03), but remain unchanged in homozygotes (Fig. 5i). The changes in COL6 expression with blood vessel size was less pronounced (adjusted *R*^2^: *APOEε*3/3: 0.67, *APOEε3/4*: 0.50, *APOEε4/4*: 0.55; all *P* values are less than 1.0 × E−15 for non-zero significance of the slopes) (Fig. 5j).

### FN1 deposition is different between demented and cognitively unaffected *APOEε4/4* carriers

**Fig. 6:**
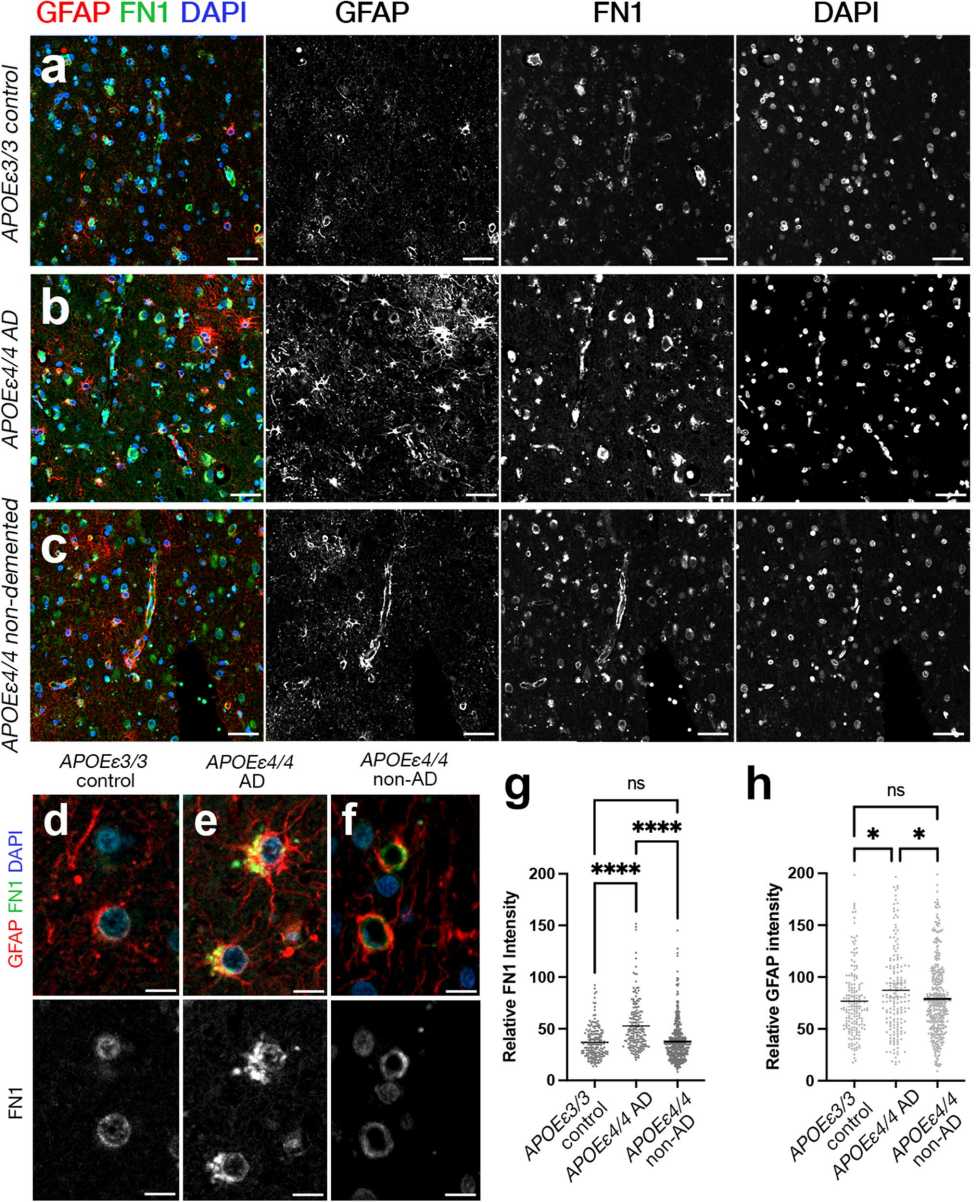
FN1 deposition and gliosis reduce to control levels in *APOEε4/4* cognitively unaffected individuals, but not in *APOEε4/4* AD patients. **a**–**c** Double IFS for FN1 (green) and GFAP (red) with DAPI nuclear counterstain in *APOEε3/3* (**a**), *APOEε4/4* AD (**b**), and *APOEε4/4* cognitively unaffected individuals (**c**). Black–white images are individual fluorescent channels for FN1, GFAP, and DAPI. **d**–**f** Two blood vessels in every condition are shown in high magnification together with FN1 channel alone. **g** FN1 intensity comparisons (2 *APOEε3/3* individuals without AD, 2 *APOEε4/4* individuals with AD, and 6 *APOEε4/4* individuals without AD). **h** GFAP intensity comparisons. Scale bars equal 50 μm (**a**-**c**) and 10 μm (**d**-**f**)

Based on our findings that FN1 deposition is increased in patients with AD and *APOE* dosage correlates with FN1 levels, we hypothesized that FN1 deposition could be a downstream driver of the pathological effects of *APOEε4* in AD. We tested this hypothesis by comparing FN1 and GFAP (marker for reactive gliosis) levels in *APOEε3/3* (control, *n* = 2), *APOEε4/4* AD (*n* = 2), and *APOEε4/4* unaffected (*n* = 6) individuals (Fig. 6, Supplementary Table 8, Supplementary Dataset 3). We found elevated reactive gliosis and FN1 deposition in *APOEε4/4* carriers with AD compared to *APOEε3/3* controls (ANOVA adjusted *P* = 1.5E−02 for GFAP intensity, 4.1E-11 for FN1 intensity) (Fig. 6). *APOEε4/4* unaffected carriers had FN1 and GFAP levels that were similar to that in controls (ANOVA adjusted *P* = 0.5245 for GFAP intensity, *P* = 0.8884 for FN1 intensity) (Fig. 6, Supplementary Fig. 3). This implies that the non-demented *APOEε4* carriers are protected from gliosis and FN1 deposition (Fig. 6g, h).

### Fibronectin loss-of-function zebrafish model enhances gliovascular endfeet retraction and microglial activity while reducing gliosis after amyloid toxicity

**Fig. 7:**
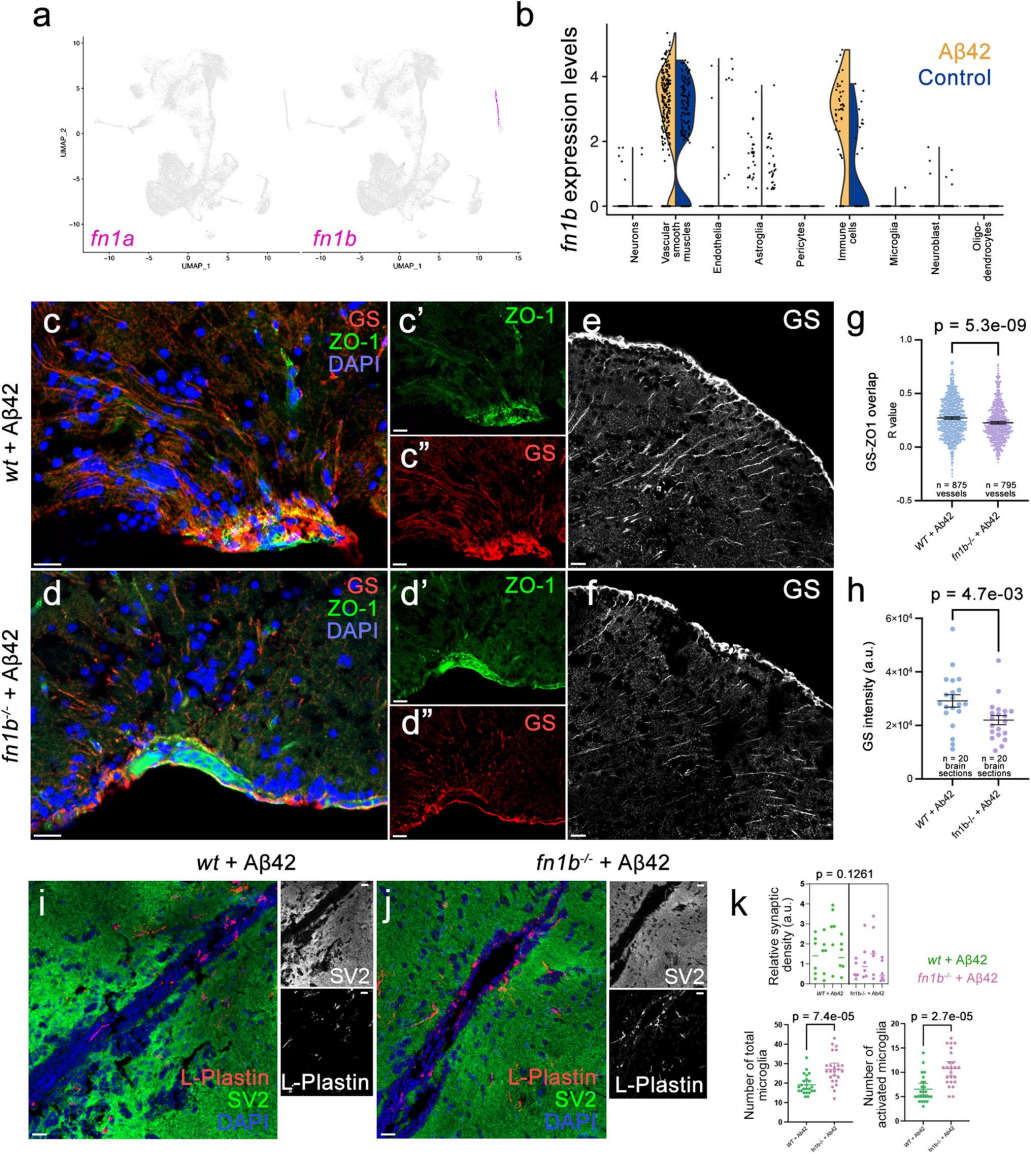
Fibronectin loss of function affects gliovascular interactions, gliosis, and microglial activity after amyloid toxicity in zebrafish brain. **a** Feature plots for fibronectin 1a (*fn1a*) and fibronectin 1b (*fn1b*) genes in zebrafish brain. **b** Violin plots in control and Aβ42-treated brains. *fn1b* is mainly expressed in vascular smooth muscle cells and immune cells and is upregulated with Aβ42. **c, d** Double IF for astroglia marker glutamine synthase (GS, red) and tight junction marker (ZO-1, green) in wild-type and *fn1b*^−/−^ animals. Individual fluorescent channels in **c′**, **c′′**, **d′**, and **d′′**. **e, f** Individual GS channels. **g** Quantification for colocalization of ZO-1 and GS. **h** Comparison of intensity measurements for GS. **i, j** Double IF for synaptic marker SV2 (green) and microglial marker l-Plastin (red) in wild-type and *fn1b*^−/−^ animals treated with Aβ42. Individual fluorescent channels in black–white channel. **k** Quantifications for synaptic density, total number of microglia, and activated microglia. Scale bars equal 25 µm

To determine whether fibronectin activity is related to cellular responses after amyloid toxicity, we used our established amyloid toxicity model in the adult zebrafish brain [11, 13, 22, 42, 47, 72]. Zebrafish has two fibronectin 1 genes: *fn1a* and *fn1b *[78]. Our single-cell transcriptomics analyses in the zebrafish brain showed that *fn1b*, but not *fn1a* is expressed in the zebrafish forebrain (Fig. 7a). *fn1b* expression is predominantly detected in vascular smooth muscle cells and immune cells, while endothelia and astroglia express *fn1b* at considerably lower levels (Fig. 7b). Amyloid toxicity results in increased *fn1b* expression in immune cells and vascular smooth muscle cells (Fig. 7b), similar to what we observed in AD brains (Figs. 5, 6). To determine the effects of fibronectin function in amyloid-induced pathology, we used an *fn1b* full knockout zebrafish line (*fn1b*^−/−^), which was previously functionally validated [33]. After treating wild-type and *fn1b*^−/−^ animals with Aβ42, we performed immunohistochemical stainings for astroglia (red, GS) and tight junctions that mark vascular structures (green, ZO-1) (Fig. 7c–f, Supplementary Dataset 4). Compared to wild-type animals treated with Aβ42, *fn1b*^−/−^ animals with Aβ42 showed less colocalization of GS and ZO-1 (− 16.3%, *P* = 5.3E−09), suggesting that gliovascular interactions were reduced with fibronectin loss of function (LOF) (Fig. 7g). Based on our previous findings that reduced gliovascular contact upon amyloid toxicity is a protective mechanism through enhancing clearance of toxic protein aggregates and immune systems activity [47], our results suggest that fibronectin could negatively regulate amyloid beta clearance and therefore an LOF variant could be protective against disease pathology. By performing intensity measurements for astroglia with GS immunoreactivity, we observed that GS intensity reduces with *fn1b* LOF (− 24.7%, *P* = 4.7E−03; Fig. 7h, Supplementary Dataset 5), indicative of reduced gliotic response upon Aβ42. To determine the effect of fibronectin on synaptic density and the number and activation state of microglia, we performed immunostainings (Fig. 7i, j, Supplementary Dataset 6) and found that loss of fibronectin leads to increased numbers of total (41.5%, *P* = 8.7E−04) and activated microglia (64.3%, *P* = 2.9E−04). We did not observe change in the synaptic density when Aβ42-treated *fn1b*^−/−^ was compared to Aβ42-treated wild-type animals (Fig. 7i–k, Supplementary Dataset 7).

## Discussion

**Fig. 8:**
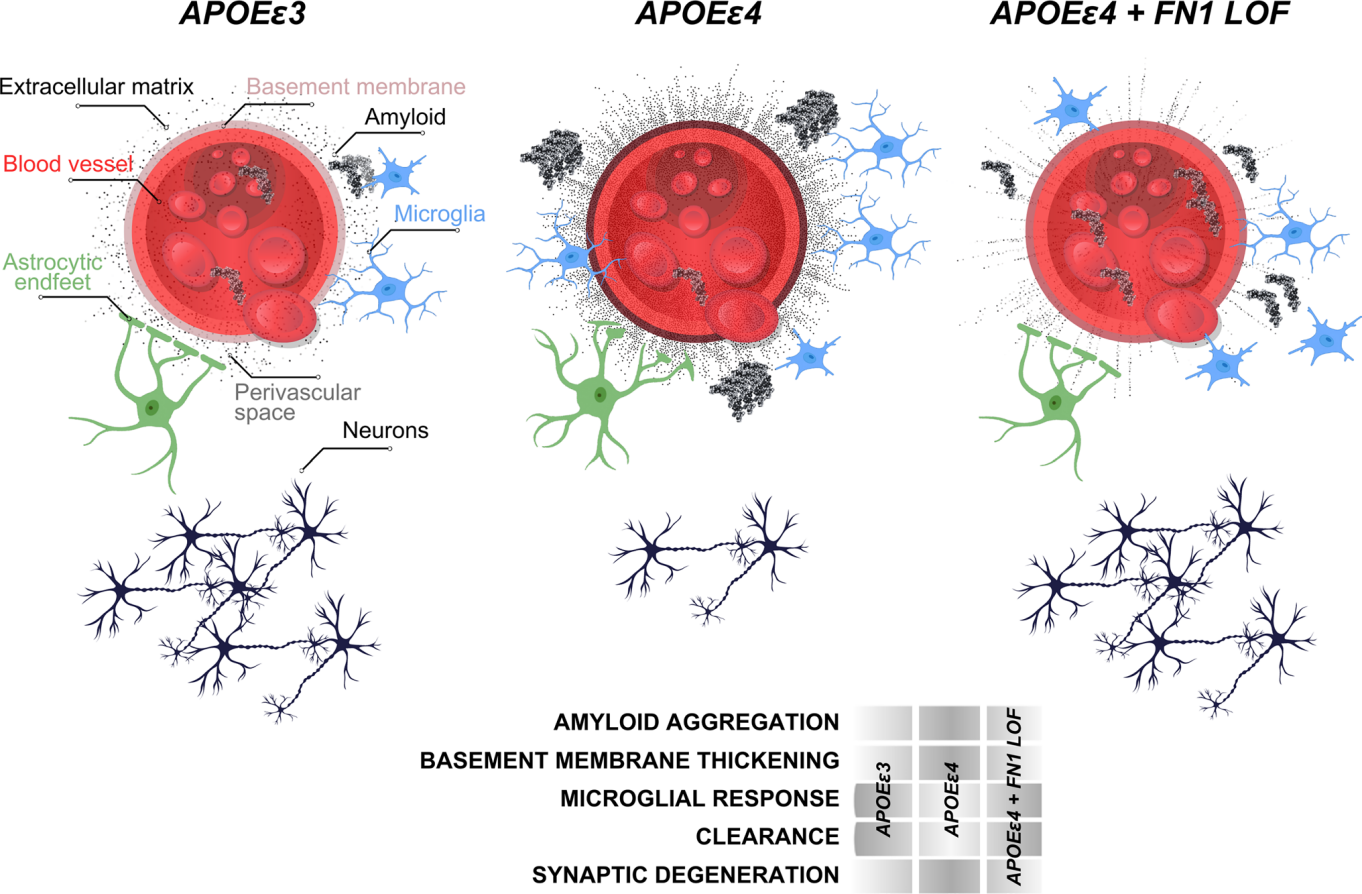
Schematic abstract for the protective effect of *FN1* variants

In our study, we found that two missense, potential loss-of-function (LOF) variants in *FN1* may protect against *APOEε4*-mediated AD pathology. We base our conclusions on four main observations: (1) *FN1* coding variants were present in cognitively unaffected *APOEε4* homozygous carriers, but not in affected carriers with clinically diagnosed AD (Supplementary Table 1), and the protective effect was independently replicated is a large cohort of *APOE*ε4 homozygous carriers. (2) Deposition of FN1 at the BBB basement membrane increases with APOEε4﻿ dosage (Fig. 5). (3) Unaffected/resilient homozygous *APOEε4* carriers above the age of 70 without AD have FN1 deposition levels similar to *APOEε3* control individuals (Fig. 6). (4) In the zebrafish brain, knockout of *fn1b* alleviates amyloid toxicity-related pathological changes (Fig. 7). These results suggest that the basement membrane thickening and remodeled ECM composition in the BBB may be a pathological contribution to *APOEε4*-mediated AD pathology that may be mitigated by variants in *FN1* or other ECM genes (Fig. 8). This conclusion is supported by the presence of variants in other BBB-related ECM components, such as *LAMA1*, *LAMA3*, and *HSPG2*, in unaffected elderly *APOEε4* carriers but not in carriers with AD (Supplementary Table 1). Therefore, our findings propose a new direction for potential therapeutic interventions reducing the impact of *APOEε4*-mediated risk of AD by targeting the BBB basement membrane. Thus, we propose that fibronectin loss of function may be a protective mechanism for AD (Fig. 8).

*APOEε4* has been associated with increased neuroinflammation and neurodegeneration, which can accelerate the progression of AD [63]. Our results in zebrafish *fn1b* knockout model showed that reduced fibronectin 1 increased the gliovascular (GV) endfeet retraction and reduced gliosis. We previously showed that the relaxed GV contact was a beneficial response to amyloid toxicity [47] as it helps enhance the clearance of toxic aggregates through the bloodstream. Additionally, gliosis is an immediate response in astroglia to insult and prevents functional restoration of neuronal activity in disease [16, 29, 73, 93]. Independent reports showed that astrocytic removal of *APOE* protects against vascular pathology [89], and gliosis is a mediator of amyloid-dependent tauopathy in late AD [6]. We propose that the relationship of fibronectin with these processes is pathogenic, and reduced fibronectin could be protective by allowing more efficient clearance through the bloodstream and reduced astrogliosis. The enhanced microglial activity supports this hypothesis, as acute activation of microglia is a beneficial response to toxic protein aggregation [36, 62].

Our results are consistent with the previous findings on *APOE*-dependent vascular pathologies and their relationship to AD [38, 45, 46, 56, 67, 79]. Endothelial fibronectin induces disintegration of endothelial integrity and leads to atherosclerotic vascular pathologies [1, 18, 95], supporting our findings that reduced fibronectin 1 protects the blood–brain barrier integrity disrupted by *APOEε4*. Our findings are coherent with the previous observations, where AD-related changes in collagen and fibronectin around the blood–brain barrier (BBB) and alterations in the BBB's structure and function were documented [43, 80, 92]. Additionally, the serum levels of fibronectin increase in AD patients in comparison to healthy individuals [15]. Collagen and fibronectin can also be early pathological markers of AD [48], where the increase in the deposition and cross-linking of basement membrane around the cerebral blood vessels lead to a thickening of the basement membrane, potentially compromising its permeability and function [35, 67, 83, 84, 90]. Fibronectin expression levels in brain vasculature increases in AD [22, 41, 49, 79, 88], where remodeling of the BM and replacing ECM with FN1 have been suggested to indicate hypoperfusion and atherosclerosis-prone state [1, 46, 54]. Additionally, *APOEε4* might regulate BM remodeling through inhibition of pericyte-mediated matrix proteinase expression [55]. Pericyte degeneration, mural cell dysfunction, and alterations in cerebrospinal flow dynamics are long-term consequences of vascular pathologies in aging and AD and is accelerated with *APOEε4* [5, 25, 27, 34, 38, 66]. Therefore, based on our findings, we propose that excess ECM deposition and BM thickening with collagen and fibronectin could promote the blood–brain barrier breakdown. Potential loss-of-function variants in ECM genes are likely to render ECM components non-functional, thus protecting against AD progression. Stronger instructive interactions of collagen and fibronectin with their receptors on various BBB cell types in AD [39, 59, 79, 88] support this hypothesis. Consistently, FN1 provides attachment surface for immune cells, which—when becomes chronic—damages the vascular functions, contribute to BBB breakdown, and loss of synaptic integrity.

We found that despite their *APOEε4/4* status, unaffected/resilient individuals who do not develop cognitive decline have lower FN1 deposition and gliosis at the vascular basement membrane that are not different from *APOEε3/3* control individuals, but significantly lower than those in *APOEε4/4* AD patients (Fig. 6). This demonstrated that FN1 is a critical component of *APOEε*4-mediated development of AD, and a yet unknown protective mechanism against the effects of *APOEε4/4* genotype suppresses FN1 deposition. We propose that FN1 is a critical downstream effector of *APOEε4* and reduced FN1 levels, either through rare, protective genetic variations in *FN1* or through other resilience mechanisms, promoting protection against AD. An interesting future research could investigate the other rare protective variants of *APOE* such as *APOEε2* [28, 31] and *APOEε3* Christchurch [70] and their effects on the BBB basement membrane.

The strength of this study is the cross-species design with pathological and functional validation to show that ECM component fibronectin could be related to key pathological aspects of AD such as toxic protein clearance, blood–brain barrier integrity, and microglial activity. We present the first knockout zebrafish for fibronectin 1 in relation to amyloid toxicity and identified cellular changes that relate to fibronectin activity.

Further studies could address some limitations of our study. First, the mechanism by which *APOEε4* enhances FN1 requires further investigations. Although in human and zebrafish brains, fibronectin is upregulated, the longitudinal relationship of amyloid aggregation to FN1 activity needs to be analyzed. Additionally, our genetic studies were conducted in clinically assessed individuals, and given the rarity of the *FN1* mutation, we did not have neuropathological assessments of *APOEε4/4* individuals with this rare protective mutation. Future studies in large-scale neuropathologic cohorts are necessary to demonstrate the pathological consequences of the rare *FN1* variants. Finally, mechanistic studies of *FN1* with and without the rare mutation are necessary to demonstrate the nuanced functional consequences.

## Materials and methods

### Ethics statement

All human samples were de-identified and the researchers could not infer or obtain personal information of the donors. Institutional Review Board approval from Columbia University Irving Medical Center and Mayo Clinic was taken before clinical data generation. Human cohorts and their characteristics are provided below. Animal experiments were carried out in accordance with the animal experimentation permits of the Institutional Animal Care and Use Committee (IACUC) at Columbia University (protocol number AC-AABN3554). Animals were maintained according to the Institutional Animal Care and Use Committee (IACUC) standards of the Institute of Comparative Medicine at the Columbia University Irving Medical Center and the accepted guidelines [2, 30, 44, 77]. The animal care and use program at Columbia University is accredited by the AAALAC International and maintains an Animal Welfare Assurance with the Public Health Service (PHS), Assurance number D16-00003 (A3007-01). Animal experiments were approved by the IACUC at Columbia University (protocol number AC-AABN3554). For zebrafish studies, 8- to 10-month-old wild-type AB strains or *fn1b*^−/−^ homozygous knockout fish lines of both genders were used. In every experimental set, animals from the same fish clutch were randomly distributed for each experimental condition.

### Human cohort information

NIA-AD Family Based Study (NIA-AD FBS): This study recruited multiplex families across the USA. Families were included if at least one member had a diagnosis of definite or probable Alzheimer’s disease [40, 51] with onset after age 60 and a sibling with definite, probable, or possible disease with a similar age at onset. Demographic information, diagnosis, age at onset for patients with Alzheimer’s disease, method of diagnosis, Clinical Dementia Rating Scale [37], and the presence of other relevant health problems were available for each individual. The age at onset for patients was the age at which the family first observed signs of impaired cognition. For unaffected family members, we used their age at the time of their latest examination without impairment. Each recruitment site used standard research criteria for the diagnosis of Alzheimer’s disease [51]. For deceased family members who had undergone autopsy, the results were used to determine the diagnosis. For analyses, clinical Alzheimer’s disease was defined as any individual meeting NINCDS–ADRDA criteria for probable or possible Alzheimer’s disease [51] and definite Alzheimer’s disease when CERAD pathological criteria [53] were met postmortem.

Washington Heights/Inwood Columbia Aging Project (WHICAP): WHICAP is a multiethnic, community-based, prospective cohort study of clinical and genetic risk factors for dementia. Three waves of individuals were recruited in 1992, 1999, and 2009 in WHICAP, all using similar study procedures [32, 60]. Briefly, participants were recruited as representatives of individuals living in the communities of northern Manhattan who were 65 years and older. At the study entry, each person underwent a structured interview of general health and function, followed by a comprehensive assessment including medical, neurological, and psychiatric histories, and standardized physical, neurological, and neuropsychological examinations. Individuals were followed every 18–24 months, repeating examinations that were similar to baseline. All diagnoses were made in a diagnostic consensus conferences attended by a panel consisting of at least one neurologist and one neuropsychologist with expertise in dementia diagnosis, using results from the neuropsychological battery and evidence of impairment in social or occupational function. All-cause dementia which was determined based on *Diagnostic and Statistical Manual of Mental Disorders, 4th Edition criteria* [4]. Furthermore, we used the criteria from the National Institute of Neurological and Communicative Disorders and Stroke–Alzheimer Disease and Related Disorders Association to diagnose probable or possible AD [51].

Estudio Familiar de Influencia Genetica en Alzheimer (EFIGA): We used families from a different ethnic group to identify protective alleles in *APOEε4* healthy individuals. This cohort comprises participants from a group of families from the Dominican Republic, Puerto Rico, and New York. Recruitment, study design, adjudication, and clinical assessment of this cohort have been previously described [86] as were details of genome-wide SNP data, quality control, and imputation procedures of the GWAS data [68, 85]. Participants were followed every 2 years and evaluated using a neuropsychological battery [76], a structured medical and neurological examination, and an assessment of depression [65, 75]. The Clinical Dementia Rating Scale (CDR) [57, 58] and functional status were done and the clinical diagnosis of Alzheimer’s disease was based on the NINCDS–ADRDA criteria [10, 50].

### Whole-genome sequencing and quality control

The demographics of the individuals selected for sequencing is shown in Table 1. WGS was performed at the New York Genome Center (NYGC) using 1 µg of DNA, an Illumina PCR-free library protocol, and sequencing on the Illumina HiSeq platform. We harmonized the WGS and the EFIGA families (*n* = 307), and jointly called variants to create a uniform, analysis set. Genomes were sequenced to a mean coverage of 30×. Sequence data analysis was performed using the NYGC automated analysis pipeline which matches the CCDG and TOPMed-recommended best practices [3]. Briefly, sequencing reads were aligned to the human reference, hs38DH, using BWA-MEM v0.7.15. Variant calling was performed using the GATK best practices. Variant filtration was performed using variant quality score recalibration (VQSR at tranche 99.6%) which identified annotation profiles of variants that were likely to be real and assigns a score (VQSLOD) to each variant.

### Identification of variants segregating in healthy *APOEε4* individuals

First, we filtered high-quality rare (MAF < 0.01 in gnomAD) variants with genotype quality (GQ) ≥ 20 and depth (DP) ≥ 10. We then excluded any variant observed in *APOE* ε4 non-carriers. Within variants that segregated in *APOE*ε4 carriers, we prioritized those that were observed in at least 1% of *APOEε4* homozygous healthy elderly (≥ 70 years) and had additional support in healthy elderly (≥ 80 years) heterozygous carriers. We further prioritized variants that were absent in AD patients carrying an *APOEε4* allele. A simplified pipeline is provided in Fig. 2.

### Genotyping, amyloid administration, and tissue preparation

A previously generated *fn1b* knockout line using CRISPR–Cas9 gene editing [33] was used in homozygous form. The full deletion was genotyped as described [33]. Amyloid-β42 was administered to the adult zebrafish brain through cerebroventricular microinjection into the cerebral ventricle [13]. Euthanasia and tissue preparation were performed as per institutional ethics committee approval and international guidelines [13, 44]. 12-µm-thick cryo-sections were prepared from these brain samples using a cryostat and collected onto glass slides which were then stored at − 20 °C.

### Replication of FN1 variant

An in-depth overview of the methodology and analyses of replication datasets is provided in the Supplementary Text. The current study followed STREGA reporting guidelines. Participants or their caregivers provided written informed consent in the original studies. The current study protocol was granted an exemption by the Stanford Institutional Review Board because the analyses were carried out on “de-identified, off-the-shelf” data; therefore, additional informed consent was not required. Case–control, family-based, and longitudinal AD genetic cohorts were available through public repositories, with genetic data from high-density single-nucleotide polymorphism microarrays, exome microarrays, whole-exome (WES) and whole-genome sequencing (WGS) (Supplementary Table 4–6). These data pertained to cohorts belonging to the ADGC and the ADSP R3. We additionally used population-based data from the UKB, where we had access to health record information to derive case–control diagnoses [17].

Genetic quality control procedures for the UKB are detailed elsewhere. ADGC and ADSP genetic data underwent extensive quality control, imputation to the TOPMed reference panel (for ADGC array-based samples) [26, 81], and ancestry determination (SNPweights v2.1) [19]. Duplicated individuals were identified and their clinical, diagnostic, and pathological data, as well as age at onset of cognitive symptoms, age at examination for clinical diagnosis, age at last examination, age at death, sex, race, ethnicity, and *APOE* genotype, were cross-referenced across cohorts. Duplicate entries with irreconcilable phenotypes were excluded. *APOE* genotypes were adjudicated using state-of-the-art *APOE* prioritization approaches, filtering out samples where *APOE* genotypes lacked robustness (prioritizing *APOE* genotypes from sequencing data and cross-referencing *APOE* genotypes from high-quality imputation with those provided in study demographics through various protein-based and DNA-based methods) [8].

Finally, in all datasets, samples were filtered to ages 60 years and above, cases or controls, belonging to non-Hispanic White ethnicity and European ancestry, and retaining only a single individual per cryptic relatedness cluster (determined down to third-degree relatedness). Secondary analyses evaluated associations with AD age at onset. Significant discoveries were considered at *P* < 0.05. All statistical analyses were conducted using R (v.4.2.1).

### Immunohistochemistry

Postmortem human brain sections from BA9 prefrontal cortex were obtained from New York Brain Bank at Columbia University and Mayo Clinic Jacksonville as paraffin-embedded blanks and with neuropathology assessments (Supplementary Table 7 and 8). Immunohistochemistry (IHC) was performed as described [47, 87]. As primary antibodies FN1 (Proteintech, catalog number 66042-1-Ig, 1:250), CD31 (Abcam, catalog number ab134168, 1:250), COL6A2 (Thermo Fisher, catalog number PA5-65085, 1:200), COL4 (Thermo Fisher, 14-9871-82, 1:100), and GFAP (Thermo Fisher, catalog number OPA1-06100), and as secondary antibodies goat anti-mouse Alexa Fluor 448 (Thermo Fisher, catalog number A-21131, 1:500) and goat anti-rabbit Alexa Fluor 555 (Thermo Fisher, catalog number A-21137, 1:500) were used. In short, for deparaffinization and hydration, xylene and alcohol were used. Antigen retrieval was performed with citrate buffer (pH: 6.0) or antigen retriever EDTA buffer (pH:8.5) in a pressure cooker or microwave for 18–25 min. Sections were blocked in 10% normal goat serum for 1 h at room temperature and were incubated with primary antibody combinations (FN1-CD31, COL6A2-COL4 or FN1-GFAP) overnight at 4 °C in a humidified chamber. Each secondary antibody to the respective primaries was applied for 2 h at room temperature. Slides were covered by mounting medium with nuclear counterstain DAPI (Thermo Fisher, catalog number D1306, 5 ng/ml). Immunohistochemistry for zebrafish was performed as described [13]. In short, the slides were dried at room temperature for 30 min and washed with PBS with 0.03% Triton X-100 (PBSTx). Primary antibody combinations (ZO-1 + GS and SV2A + l-Plastin) were applied overnight at 4 °C. Next day, after washing three times with PBSTx appropriate secondary antibodies were applied for 2 h at room temperature. The slides were then washed several times before mounting using 70% glycerol in PBS. The following antibodies were used: mouse anti-ZO-1 (1:500, Thermo Fisher Cat. No. 33-9100), rabbit anti-glutamine synthetase (GS) (1:500, Abcam Cat. No. ab176562), mouse anti-SV2A (1:500, DSHB Cat. No. SV2), and rabbit anti-l-plastin (1:3000, gift from Michael Redd), secondary antibodies goat anti-mouse Alexa Fluor 448 (Thermo Fisher, catalog number A-21131, 1:500), and goat anti-rabbit Alexa Fluor 555 (Thermo Fisher, catalog number A-21137, 1:500). For antigen retrieval of ZO-1 and SV2, slides were heated in 10 mM sodium citrate (pH:8.0) at 85 °C for 15 min before primary antibody incubation.

### Image acquisition, quantification, and statistical analyses

Five random illumination field images per patient from the immunostained slides were acquired using Zeiss LSM800 confocal microscope equipped with ZEN software (version blue edition, v3.2, Carl Zeiss, Jena, Germany). Based on vascular markers, coronally sectioned blood vessels were delineated with the selection tool of ZEN software. Fluorescence intensity measures, diameter, and area was calculated. Acquisitions were performed in a blinded fashion (sample IDs, neuropathology details, and genotypes were revealed after the acquisition) and no vessels were specifically left out unless their diameters were larger than 50 μm. GraphPad Prism software version 9.2.0. was used for the statistical analyses. For multiple comparisons, one-way Brown–Forsythe and Welch ANOVA test with two-stage linear step-up procedure of Benjamini, Krieger, and Yekutieli comparison with individual calculation of variances was used. For non-Gaussian distributions, non-parametric Kruskal–Wallis test with Dunn’s multiple comparison test was performed. For correlation of vessel diameter to fluorescent intensity, simple linear regression model and second-order polynomial robust regression with no weighting was used. Significance is indicated by ∗(*P* < 0.0332), ∗∗(*P* < 0.0021), ∗∗∗(*P* < 0.002), ****(*P* < 0.0001). No asterisks indicate non-significance. No sample set was excluded from the analyses unless the histological sections were damaged severely during the acquisition of the sections (constitutes less than 3% of all sections analyzed).

For zebrafish studies, the effect sizes for animal groups were calculated using G-Power, and the sample size was estimated with n-Query. Four zebrafish from both sexes were used per group. For quantification of SV2-positive synapses, 3D object counter module of ImageJ software was used with the same standard cutoff threshold for every image. For quantification of activated/resting l-plastin-positive microglial cells, two different microglial states were classified based on their cellular morphology: slender and branched as resting microglia; round and regular as active microglia. Six images each from telencephalon sections were analyzed per animal. For colocalization studies, vascular fields were determined using ZO-1 staining on sections (20 for every group), and colocalization with glial endfeet labeled with GS stainings was performed using ImageJ software (v.2.1.0/1.53c) with its colocalization test. Data acquisition was randomized with Fay (*x, y, z* translation) to acquire in total 1670 data points from two experimental groups. R(and) correlation values from wild-type and *fn1b*^−/−^ animals were compared using GraphPad Prism (v.9.2.0). Intensity values for individual fluorescent channels were obtained with modal gray values and integrated density measurements using ImageJ. Comparison of 40 sections from two experimental groups was performed. An unpaired non-parametric Kolmogorov–Smirnov *t* test was performed to test the statistical significance for all analyses.

### *In silico* structure prediction

Protein structures, interspecies similarities, and the deleterious effects of variants were analyzed by SWISS-MODEL protein structure homology-modeling server through Expasy web server (https://swissmodel.expasy.org). SWISS-MODEL repository entries for respective proteins were retrieved and compared to desired protein orthologs using the superposition function. Deleterious mutation prediction was performed using Ensembl-integrated AlphaFold prediction model with SIFT, MetaLR, and REVEL modules for prediction of deleteriousness.

### Amyloid toxicity and single-cell sequencing

Amyloid toxicity was induced as described [13, 47] in the adult telencephalon; the brains were dissected and single-cell suspensions were generated as previously described [23, 24]. Chromium Single-Cell 3’ Gel Bead and Library Kit v3.1 (10X Genomics, 120,237) was used to generate single-cell cDNA libraries. Generated libraries were sequenced via Illumina NovaSeq 6000 as described [12, 13, 23, 24, 71]. The cell clusters were identified using a resolution of 1. In total, 34 clusters were identified. The main cell types were identified by using *s100b* and *gfap* for astroglia; *sv2a, nrgna, grin1a, grin1b* for neurons; *pdgfrb* and *kcne4* for pericytes; *cd74a* and *apoc1* for microglia; *mbpa* and *mpz* for oligodendrocytes; *myh11a* and *tagln2* for vascular smooth muscle cells, *kdrl* for endothelial cells.

### Supplementary Information

Below is the link to the electronic supplementary material.

Supplementary file1 (DOCX 199 KB)

Supplementary Figure 1: Structure and deleteriousness prediction for FN1 and COL6A2 (TIF 6441 KB)

Supplementary Figure 2: Individual breakdown of FN1 intensity comparisons in Figure 5d (TIF 2852 KB)

Supplementary Figure 3: Individual breakdown of FN1 intensity comparisons in Figure 6h (TIF 2059 KB)

Supplementary Table 1: The list of coding variants in Hispanics. Supplementary Table 2: The list of coding variants in non-Hispanic Whites. Supplementary Table 3: The list of SNPs. Supplementary Table 4: Overview of genotyping platforms across all available AD-related genetic data from ADSP and NACC. Supplementary Table 5: Overview of ADSP studies with WES or WGS available through NIAGADS DSS (NG00067). Supplementary Table 6: Case-control regression sensitivity analyses for rs140926439 in APOEε4∕4 carriers. To ensure an independent replication of discovery findings, in ADGC, samples from NIA-LOAD were excluded, while ADSP whole genome sequencing data was fully excluded. Combined results reflect a fixed-effects inverse-variance weighted meta-analysis. Supplementary Table 7: Demographics and APOE genotypes of the post-mortem human brains used for the study. Supplementary Table 8: Demographics and *APOE* genotypes of the post-mortem human brains used for vascular deposition of FN1 (XLSX 526 KB)

Supplementary Dataset 1: Total fluorescent intensity values for COL4, COL6A and DAPI, according to *APOE* genotype. Supplementary Dataset 2: Total fluorescent intensity values for FN1, CD31 and DAPI, according to *APOE* genotype. Supplementary Dataset 3: Total fluorescent intensity values for FN1, GFAP and DAPI, according to *APOE* genotype and Alzheimer’s disease status. Supplementary Dataset 4: Fluorescent intensity for overlapping GS (astrocytes) and ZO-1 (tight junctions) in wild type and *fn1b*^-/-^ animals treated with Aβ42. Supplementary Dataset 5: Fluorescent intensity for ventricular GS (astrocytes) in wild type and *fn1b*^-/-^ animals treated with Aβ42. Supplementary Dataset 6: Number of microglia and their activation state in wild type and *fn1b*^-/-^ animals treated with Aβ42. Supplementary Dataset 7: Automated quantification results of synaptic puncta in wild type and *fn1b*^-/-^ animals treated with Aβ42 (XLSX 420 KB)

## Data Availability

Whole Genome Sequencing data (WGS) from EFIGA and NIA-AD FBS cohorts that were analyzed in the study have been deposited to NIAGADS (The National Institute on Aging Genetics of Alzheimer’s Disease Data Storage Site) and are shared with the research community as part of the Alzheimer’s Disease Sequencing Project (ADSP). The brain tissue used in this analysis was requested from the New York Brain Bank at Columbia University. Raw data generated from the brain tissue and zebrafish experiments are available upon request. The zebrafish gliovascular single-cell dataset can be accessed at NCBI’s Gene Expression Omnibus (GEO) with the accession number GSE225721.
