# Supplementary figures and images for: Rare genetic variation in fibronectin 1 (FN1) protects against APOEε4 in Alzheimer’s disease
Source: Acta Neuropathol. 2024 Apr 10;147(1):70. doi: 10.1007/s00401-024-02721-1 (PMC11006751; doi:10.1007/s00401-024-02721-1)

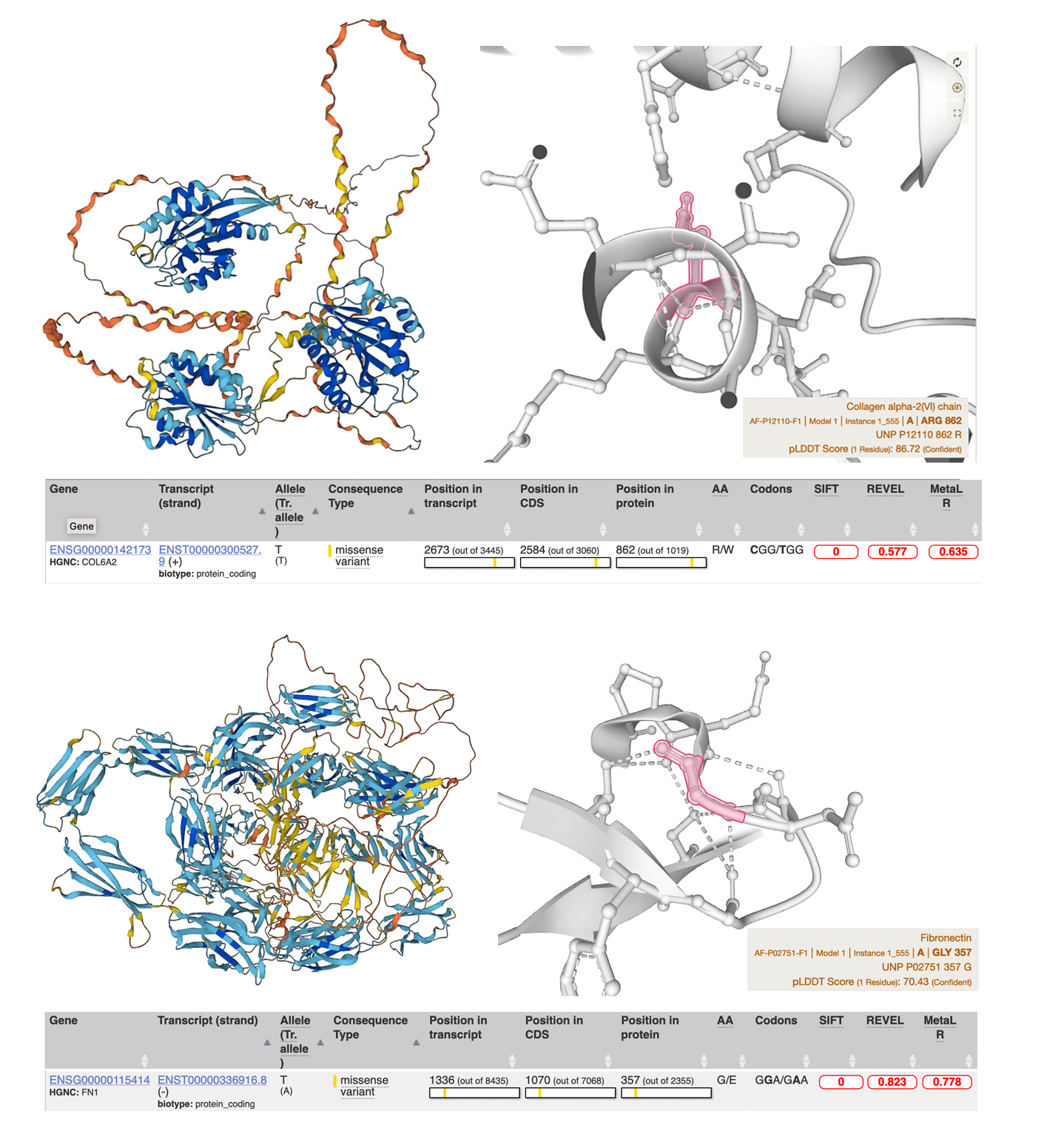

Supplement: Supplementary file 2 — Supplementary Figure 1: Structure and deleteriousness prediction for FN1 and COL6A2 (TIF 6441 KB) [file 401_2024_2721_MOESM2_ESM.tif]

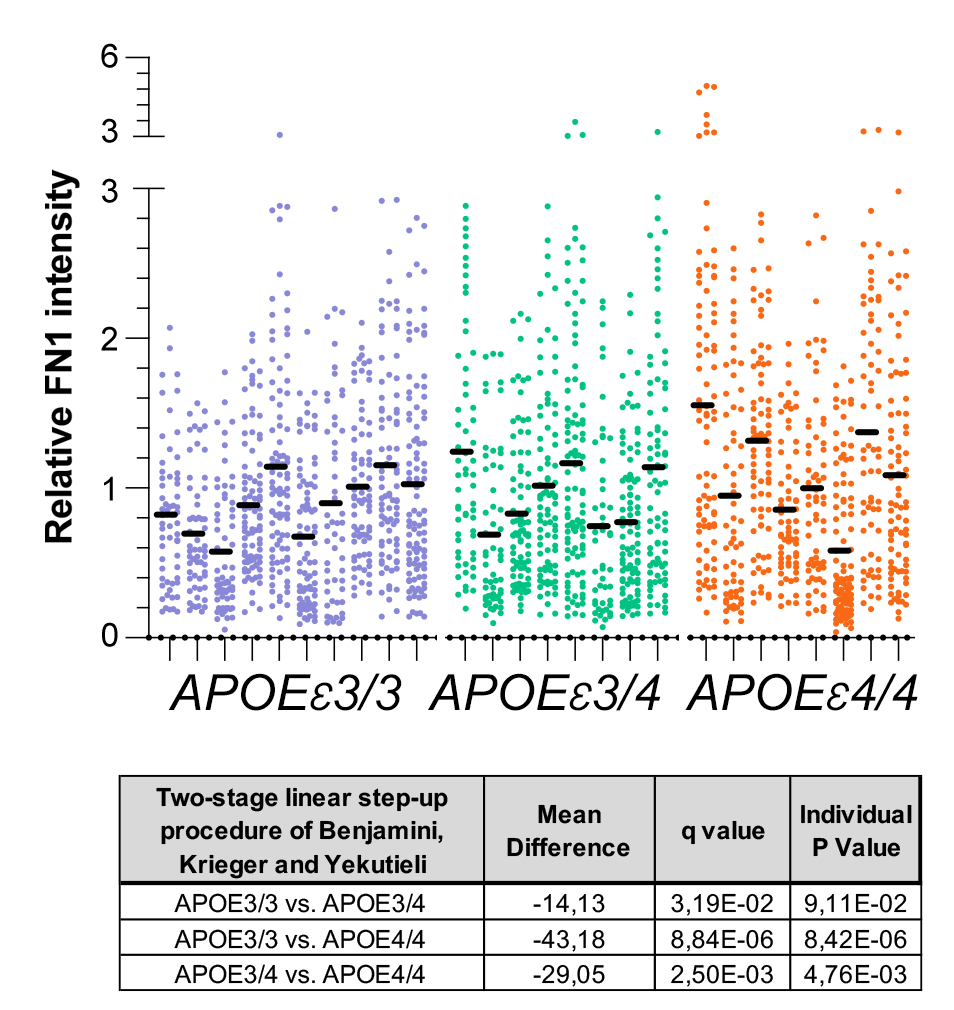

Supplement: Supplementary file 3 — Supplementary Figure 2: Individual breakdown of FN1 intensity comparisons in Figure 5d (TIF 2852 KB) [file 401_2024_2721_MOESM3_ESM.tif]

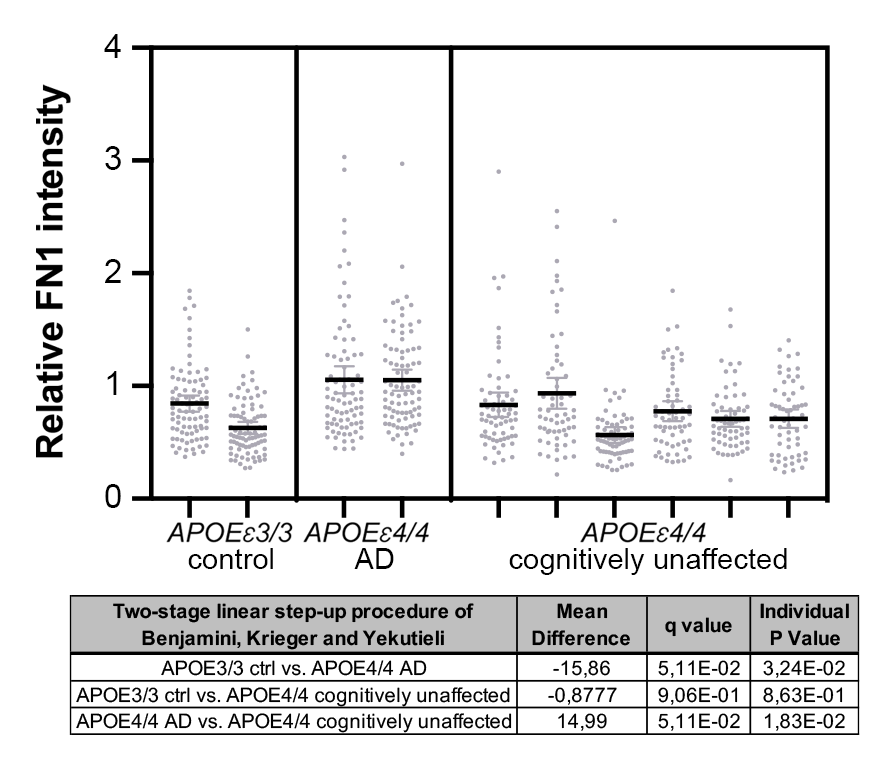

Supplement: Supplementary file 4 — Supplementary Figure 3: Individual breakdown of FN1 intensity comparisons in Figure 6h (TIF 2059 KB) [file 401_2024_2721_MOESM4_ESM.tif]
